# Relationship between inflammatory markers, glycated hemoglobin and placental weight on fetal outcomes in women with gestational diabetes

**DOI:** 10.20945/2359-3997000000099

**Published:** 2019-02-01

**Authors:** Fernanda Oliveira Braga, Carlos Antonio Negrato, Maria de Fátima Bevilacqua da Matta, João Régis Ivar Carneiro, Marília Brito Gomes

**Affiliations:** 1 Universidade do Estado do Rio de Janeiro Universidade do Estado do Rio de Janeiro Unidade de Diabetes Rio de Janeiro RJ Brasil Unidade de Diabetes, Universidade do Estado do Rio de Janeiro (UERJ), Rio de Janeiro, RJ, Brasil; 2 Associação dos Diabéticos de Bauru Bauru SP Brasil Associação dos Diabéticos de Bauru, Bauru, SP, Brasil; 3 Universidade Federal do Rio de Janeiro Universidade Federal do Rio de Janeiro Departamento de Nutrologia Rio de Janeiro RJ Brasil Departamento de Nutrologia, Universidade Federal do Rio de Janeiro (UFRJ), Rio de Janeiro, RJ, Brasil

**Keywords:** Gestational diabetes, inflammation, cytokines, placenta, macrosomia, preterm birth

## Abstract

**Objective:**

The aim of this study was to evaluate the relationship between inflammatory cytokines, placental weight, glycated hemoglobin and adverse perinatal outcomes (APOs) in women with gestational diabetes mellitus (GDM).

**Subjects and methods:**

This was a prospective, longitudinal and observational study conducted from April 2004 to November 2005 in Bauru, Brazil. Included patients had singleton pregnancies and performed a 100 g OGTT and had the levels of C-reactive protein (CRP), interleukin (IL)-6, TNF alfa and glycated hemoglobin (HbA1c) determined at 24-28^th^ gestation weeks.

**Results:**

A total of 176 patients were included, of whom 78 had the diagnosis of GDM (44.3%). Multivariate analysis demonstrated that HbA1c, age, body mass index (BMI) and previous history of GDM were independent predictors for GDM diagnosis. ROC curve indicated that HbA1C levels ≥ 5.1% at 24-28 weeks gestation were associated with GDM. No difference was found in IL-6, tumor necrosis factor alpha (TNF-alpha) and CRP serum levels in women with and without GDM. Multivariate analysis showed that placental weight was significantly associated with APOs (p < 0.005), with a cut-off value of 610 grams as demonstrated by the ROC curve.

**Conclusion:**

Placental weight ≥ 610 grams and HbA1C ≥ 5.1% were found to be associated with APOs and GDM, respectively, and their evaluation should be part of prenatal care routine.

## INTRODUCTION

Gestational diabetes mellitus (GDM) is the most common metabolic disorder found during gestation and is defined as hyperglycemia of variable severity with onset or first recognition during pregnancy that does not clearly characterize any type of preexisting diabetes (1). In Brazil, a study conducted in the 90's called Brazilian Gestational Diabetes Study Group has found that approximately 7.6% of pregnancies were complicated by GDM (2). In 2010, the International Association of Diabetes and Pregnancy Study Groups has proposed new diagnostic criteria for the diagnosis of GDM with lower cutoff values, which lead to higher diagnostic rates (3). If these criteria were to be used in the population that was evaluated in the Brazilian Gestational Diabetes Study Group, the prevalence of GDM would be 18% instead of 7.6% as previously found (4). This finding shows that the prevalence of GDM varies widely depending on the diagnostic criteria that are used (5).

GDM is a heterogeneous disorder, resulting from an interaction between genetic and environmental risk factors. Currently, one of the most important risk factors for the development of GDM is the increasing prevalence of overweight/obesity which is present in up to 60.0% of women on reproductive age in the USA and some other developed countries (5).

GDM is associated with a constellation of alterations such as impaired insulin secretion, hyperinsulinemia, insulin resistance, obesity, dyslipidemia and hypertension (6), that are related to an increased risk of adverse perinatal outcomes (APOs) such as large for gestational age babies (LGA), overweight (ponderal index) and low Apgar scores (< 7) (7).

In general, specific risks of poorly controlled diabetes in pregnancy, that can be evaluated by HbA1c levels, include spontaneous abortion, fetal malformations, preeclampsia, intrauterine fetal demise, macrosomia, neonatal hypoglycemia and neonatal hyperbilirubinemia. Diabetes in pregnancy may increase the risk of obesity and type 2 diabetes in the offspring later in life (8).

Placenta is a maternal-fetal organ that separates the maternal and fetal circulations and plays a central metabolic role in pregnancy, mainly in fetal development (9). Several complications found in pregnancy such as GDM, preeclampsia, intrauterine growth restriction, prematurity and low birth weight are linked to angiogenic placental processes that are related to low and high placental weight (10). The human placenta expresses several cytokines including tumor necrosis factor-alpha (TNF-alpha), resistin, C-reactive protein (CRP), interleukin 6 (IL-6) and leptin which are also produced by adipose cells. Cytokines are produced by three different placental cell types: Hofbauer cells, trophoblast cells and vascular endothelium cells. Leptin and IL-6 are released into the fetal and maternal systemic circulation; they can exert endocrine actions by acting at remote sites from their original production site. In contrast, TNF-alpha is poorly released from placenta and hence is more likely to exert paracrine effects. In GDM, the overexpression of placental TNF-alpha is associated with increased fetal adiposity (11).

The aim of this study was to evaluate the relationship between inflammatory markers, placental weight, glycated hemoglobin and APOs in women with GDM.

## SUBJECTS AND METHODS

This was a prospective, longitudinal and observational study conducted between April 2004 and November 2005 in Bauru, a Southeastern Brazilian city. The methodology has been described previously (7). All patients received free health-care from the Brazilian National Health Care System (BNHCS). One hundred and eighty women with singleton pregnancies were invited to participate. In the 3^rd^ trimester (24-28^th^ weeks of gestation) a 100g OGTT was performed; the cutoff values for the OGTT were those proposed by Carpenter & Coustan (12). After performing the test, patients were classified as having GDM if they presented at least two altered values in the curve or controls if they had a normal test (CG).

This study was approved by the ethics committee of Botucatu's School of Medicine – São Paulo State University (Unesp), Brazil. Written informed consent was obtained from all patients.

The following maternal variables were accessed using a questionnaire during a clinical visit: age, parity, ethnicity, years of school attendance, family income (Brazilian minimum wage), weight and length at birth, pre-pregnancy body mass index (BMI), family's history of diabetes, hypertension, obesity and dyslipidemia. At the screening, weight, height, legs length, blood pressure, waist circumference (at the point of minimal abdominal girth) and hip circumference (at the point of maximum extension of the buttocks) were measured. BMI was calculated dividing weight in kilograms by the square of the height in meters. A prepregnancy BMI ≥ 30 kg/m^2^ was defined as obesity. When a systolic blood pressure > 140 mmHg or a diastolic blood pressure > 90 mmHg was found on at least two occasions, at least six hours apart, the diagnosis of hypertension was made. The waist-to-hip ratio was calculated dividing waist by hip circumferences.

At the time the OGTT was made, blood samples were collected to determine fasting levels of glucose, HbA1C, total cholesterol, HDL, LDL, VLDL cholesterol and triglycerides. Sera samples were stored at −80°C. Glucose oxidase method (Glucose–analyzer II Beckman, Fullerton, CA, USA) was used to determine blood glucose levels. Triglycerides, cholesterol and its fractions (LDL, VLDL, HDL) were measured by enzymatic colorimetric assays. High performance liquid column method (HPLC) was used to determine HbA1C values (Dia-Stat analyzer, Bio-Rad Laboratories, Hercules, CA, USA reference values: 4.0-6.3%).

The following biochemical parameters were measured: CRP by turbidimetric method (A25 BioSystems and CRP kit BioSystems, with a reference value of < 0.3 mg/dl and an intra-assay and inter-assay coefficient of variation of 1.8% and 3.6%, respectively); IL-6 and TNF-alpha dosages by the MAGPIX (Luminex), multiplex immunoassay (xMAP Technology) with the Human Adipokine Magnetic Bead Panel 2 kit with coefficients of variation for TNF-alpha (intra-assay of 3.0% and inter-assay of 19.0%) and for IL-6 (intra-assay of 2.0% and inter- assay of 10.0%).

The following fetal data were collected: birth weight, length, ponderal index, gender, gestational age at delivery, Apgar scores at 1, 2 and 5 minutes, APOs and congenital malformations. Preterm was present when gestational age was < 37 weeks. The need for a baby to be admitted into an intensive care unit (ICU) was defined by the presence of any acute morbidity. The presence of malformations, respiratory distress syndrome, icterus, infections, LGA, macrosomia, neonatal hypoglycemia and the need to be admitted into an ICU, were considered as APOs.

Ponderal index was determined by the ratio between 100 times the weight and the cube of the length in cm. Lubchenco's classification was used to determine the relationship between newborns’ weight to gestational age (13). Placental weight was obtained immediately after delivery, in the same balance used to evaluate the newborn's weight. Weight was measured in kilograms (kg), grams (g) and in subsequent scales of up to 0.05 kg.

### Statistical analysis

Data are presented as means (±SD) or median (minimum-maximum) for continuous variables and numbers (relative frequencies) for discrete variables. Comparisons between independent continuous variables were performed using Mann-Whitney test. Chi-Square (χ^2^) or Fisher tests were used for comparisons between discrete variables. Spearman coefficient of correlation (rho) was performed between clinical and laboratory maternal and fetal data.

Multivariate stepwise forward logistic regression was performed to identify independent demographic and clinical predictors of GDM (yes or no) and for the presence or absence of APOs (LGA, macrosomia, preterm birth, and need for ICU admission). In all models the following parameters were described: coefficient (B), standard error (SE), odds ratio (OR) and 95% confidence interval (95% CI). The ROC (receiver operator characteristic) curve was constructed from these analyzes. Analyses were performed using SAS^®^ System, version 6.11 (SAS Institute, Inc., Cary, North Carolina). A two-sided *p* value less than 0.05 was considered significant.

## RESULTS

### Demographic, clinical and laboratory data according to the presence or absence of GDM

A total of 176 patients were included in the study of whom 78 (44.3%) had the diagnosis of GDM. Four patients were excluded: two were HIV positive and in two blood samples were not adequately stored.

Patients in the GDM group were older, mostly Caucasians n = 51 (65.4%), had less years of school attendance and lower income. They were also shorter and had shorter legs’ length than the CG. They were heavier and presented higher blood pressure levels (both diastolic and systolic). Pregnant women with GDM had a higher frequency of previous history of GDM, higher levels of fasting glucose and HbA1c. These data are described in Tables 1–3.

**Table 1:** Clinical, demography and laboratory data of the population according to OGTT

Variable	GDM			Normal OGTT			p value^a^
	N	Median	IQR	N	Median	IQR	
Age (years)	78	31.0	29.0-37.0	98	27.5	24-32	< 0.0001
Education level (years)	78	11.0	8.0-12.3	98	12.0	10.0-15.0	0.004
Family income (Brazilian minimum wage)	78	2.65	1.67-7.9	98	6.0	3.0-12.0	0.0001
Height (m)	78	1.61	1.58-1.67	98	1.65	1.59-1.7	0.010
Legs length (cm)	78	73.3	71.0-76.1	98	75.0	72.5-78.6	0.011
Legs/height ratio (%)	78	45.6	44.5-46.4	98	45.8	45.0- 46.7	0.089
Weight (kg)	78	70.0	62.0-87.5	98	64.0	57.8-72.0	0.0009
Body Mass Index (kg/m^2^)	78	27.8	23.6-32.1	98	22.8	20.9-27.3	< 0.0001
Systolic blood pressure (mmHg)	78	120	110-120	98	110	100-120	0.0001
Diastolic blood pressure (mmHg)	78	80	70-80	98	70	70-80	< 0.0001
Fasting glycemia (mg/dL)	72	90.5	82.6-103.3	89	74.0	67.0-79.0	< 0.0001
HbA1c (%)	72	5.68	5.09-6.10	88	4.8	4.41-5.20	< 0.0001
Total cholesterol (mg/dL)	72	209.0	191.0-238.0	89	230.0	206.0-261.0	0.001
LDL cholesterol (mg/dL)	70	112.0	89.0-141.0	89	127.0	110.0-153.0	0.008
HDL cholesterol (mg/dL)	72	56.3	47.6-66.9	89	64.0	54.3-76.7	0.002
VLDL cholesterol (mg/dL)	71	37.0	27.4-44.9	89	37.0	25.6-48.0	0.90
Triglycerides (mg/dL)	72	185.0	132.0-227.0	89	185.0	128.0-240.0	0.78
IL-6 (pg/mL)	78	0.175	0.120-0.298	98	0.155	0.100-0.325	0.77
CRP (mg/dL)	78	0.585	0.335-1.123	98	0.530	0.250-0.883	0.40
TNF alpha (pg/mL)	78	0.230	0.130-0.330	98	0.200	0,130-0.310	0.76

IQR: interquartile range; GDM: gestational diabetes mellitus; OGTT: oral glucose tolerance test; CRP: C-reactive protein; TNF alpha: tumor necrosis factor alpha; TSH: thyroid-stimulating hormone.

a Mann-Whitney test.

**Table 2:** Previous clinical and obstetric history of the population according to OGTT

Variable	Category	GDM		Normal OGTT		P value
		N	%	N	%	
Polycystic ovary syndrome	Yes No	21 57	26.9 73.1	39 59	39.8 60.2	0.073
Acanthosis	Yes No	50 28	64.1 35.9	27 71	27.6 72.4	< 0.0001
Smoking	Yes No	6 72	7.7 92.3	7 91	7.1 92.9	0.15
GDM	Yes No	13 65	16.7 83.3	6 92	6.1 93.9	0.025
Abortion	Yes No	21 57	26.9 73.1	25 73	25.5 74.5	0.83
Stillbirth	Yes No	3 75	3.8 96.2	2 96	2.0 98.0	0.39
Babies’ mal formation	Yes No	5 73	6.4 93.6	3 95	3.1 97.0	0.24
Polyhydramnios	Yes No	26 52	33.3 66.7	30 68	30.6 69.4	0.70
Multiple pregnancy	Yes No	0 78	0.0 100.0	4 94	4.1 95.9	0.094
Respiratory distress syndrome	Yes No	5 73	6.4 93.6	1 97	1.0 99.0	0.061
Preterm birth	Yes No	13 65	16.7 83.3	10 88	10.2 89.8	0.21
Neonatal hypoglycemia	Yes No	0 78	0.0 100.0	0 98	0.0 100.0	NA
Icterus	Yes No	7 71	9.0 91.0	5 93	5.1 94.9	0.31
< 2,500g newborn	Yes No	6 72	7.7 92.3	7 91	7.1 92.9	0.89
> 4,000g newborn	Yes No	9 69	11.5 88.5	11 87	11.2 88.8	0.95
Pregnancy induced hypertension	Yes No	12 66	15.4 84.6	11 87	11.2 88.8	0.42
Newborn death	Yes No	3 75	3.8 96.2	0 98	0.0 100.0	0.085
Familial DM	Yes No	65 13	83.3 16.7	72 26	73.5 26.5	0.12

OGTT: oral glucose tolerance test; GDM: gestational diabetes mellitus; DM: diabetes mellitus.

χ^2^ test or Fisher test.

**Table 3:** Fetal demographic data according to OGTT

Variable	GDM			Normal OGTT			p value^a^
	N	Median	IQR	N	Median	IQR	
Newborn weight (grams)	77	3,195	2935-3555	94	3,275	2,993-3,564	0.36
Length (cm)	77	48.0	46.5-49.0	94	48.5	47.0-50.0	0.18
Head circumference (cm)	76	35.0	33.5-35.0	93	35.0	34.0-36.0	0.24
Thoracic circumference (cm)	76	33.0	32.0-34.0	93	33.2	32.0-34.0	0.96
Waist circumference (cm)	73	32.0	30.0-33.0	78	32.0	30.0-33.0	0.72
Apgar T1 score	77	9	8-9	93	9	8-9	0.68
Apgar T2 score	77	10	9-10	93	10	9-10	0.92
Apgar T5 score	77	10	10-10	93	10	10-10	0.52
Placental weight (grams)	72	600	500-684	78	580	518-713	0.65

IQR: interquartile range; GDM: gestational diabetes mellitus; OGTT: oral glucose tolerance test.

a Mann-Whitney test.

### Multivariate Analysis according to the presence or absence of GDM

**Figure 1:**
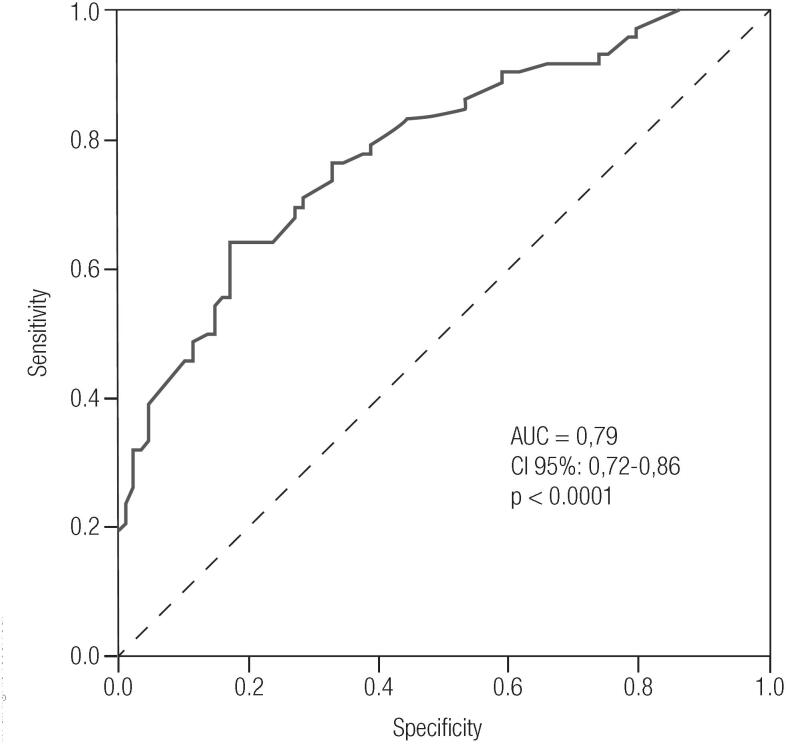
ROC curve for HbA1c as a predictor for GDM.

Multivariate logistic analysis performed with 160 observations (missing data of 16 patients for HbA1c) showed that HbA1c [OR 4.92; (95% CI 2.51-9.65); B 1.5931; SE 0.3438, p < 0.0001], age [OR 1.14; (95% CI 1.06-1.23); B 0.1341; SE 0.0368, p = 0.0002], BMI [OR 1.09; (95% CI 1.02-1.17); B 0.0880; SE 0.0351, p = 0.012] and previous history of GDM [OR 3.73; (95% CI 1.01-13.8); B 1.3162; SE 0.6669, p = 0.048] were independent predictors of GDM. Figure 1 illustrates the ROC curve of HbA1c for GDM. An area under the curve (AUC) of 0.79 [95% CI (0.72-0.86)] p < 0.0001) was observed. The cut-off value according to the ROC curve for GDM was HbA1c ≥ 5.1%, with a sensitivity of 70.8% and a specificity of 71.6%.

### Clinical and laboratory data, including inflammatory markers and multivariate analysis according to the presence or absence of APOs

There was no significant difference between the two groups in the levels of the evaluated cytokines, in APOs and in placental weight (Tables 1 and 3). We observed a correlation between levels of TNF-alpha, maternal BMI and CRP with placental weight (rho = 0.188; p = 0.021, r = 0.186; p = 0.022, r = 0.176; p = 0.031, respectively). A correlation between CRP and HbA1c levels (rho = 0.167, p = 0.035), in the total sample was also noted. An association between blood glucose [93.2 (76.0-115.4) *vs.* 79.0 (71.0-88.3) mg/dl; p < 0.003] and CRP [0.86 (0.68-1.36) mg/dl; p < 0.01] levels with prematurity was found. A correlation between CRP and HbA1c levels (rho = 0.167, p = 0.035) in the total sample was also observed.

Multivariate logistic analyses were performed with 150 observations (loss of placental weight records n = 26) with APOs’ as dependent variable. The only significant independent variable was placental weight with an [OR 0.0014; (95% CI 1.001-1.007; B 1.004; SE 0.0040, p = 0.005].

**Figure 2:**
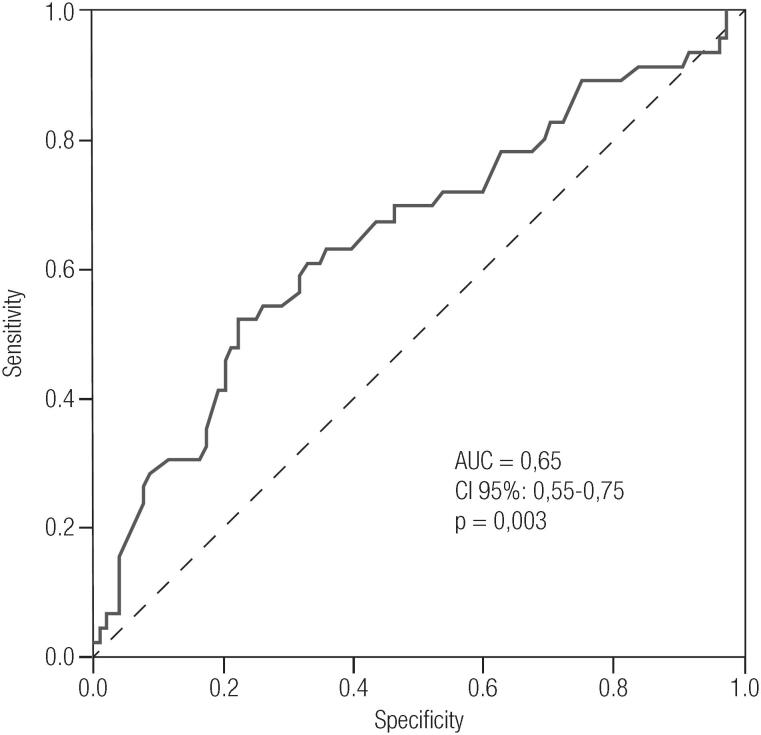
Receiver Operator Characteristics (ROC) curve for placental weight as a predictor of fetal adverse outcomes.

Figure 2 illustrates the ROC curve of placental weight for the presence or absence of APOs. An AUC of 0.65 with a 95% CI of 0.55 to 0.75, (p = 0.003) was observed. The cut-off point according to the ROC curve was placental weight ≥ 610 grams, with a sensitivity of 63.0% and a specificity of 64.4%.

## DISCUSSION

No difference was found in serum levels of IL-6, TNF-alpha, CRP and placental weight in women with GDM compared to those from the CG, between 24-28 gestation weeks. We found that values of HbA1C ≥ 5.1% at 24 to 28 weeks of gestation could be associated with GDM, however with high sensitivity and low specificity. A placental weight ≥ 610 grams was also found to be associated with the presence of APOs.

Our prevalence of GDM was higher than the prevalence found in the Brazilian Study on Gestational Diabetes (BSGD) in the 1990s, which was 7.6% (2). This may be justified by the fact that our study was performed at a secondary level medical care service, focused on diabetes in pregnancy, and also by the increasing prevalence of overweight and obesity in Brazil in recent years. In 1999, around the time this study begun, the estimated prevalence of obesity in the Brazilian general population was of 32.0% (14) and has been steadily increasing ever since, reaching 53.8% in 2016 (15). It is possible that if these data were to be collected nowadays, the rates of GDM would be even greater.

In our study, pregnant women with GDM were older, shorter, heavier, with higher blood pressure levels and shorter legs (16,17) as expected, since all these characteristics are risk factors for the development of GDM (18).

Multivariate analysis for the prediction of GDM demonstrated that HbA1c, age, BMI and previous history of GDM were independently associated with GDM. The ROC curve indicated that values of HbA1C ≥ 5.1% at 24 to 28 gestation weeks are associated with GDM.

Universal screening for diabetes in pregnancy is recommended by the International Association of the Diabetes and Pregnancy Study Groups (IADPSG) (3), the American Diabetes Association (ADA) (1) the World Health Organization (WHO) (19) and more recently by the Brazilian Diabetes Association (BDS), Brazilian Federation of Gynecologists and Obstetricians (Febrasgo), Pan American Health Organization (PAHO) and the Brazilian Ministry of Health at the first antenatal visit (20). However what should be the most appropriate test and glucose levels thresholds are still debated in many regions and countries around the world (21,22). This screening should be done with a fasting glycemia and a 75g OGTT at the first antenatal visit or at 24-28 gestation weeks, respectively (20).

The OGTT is time consuming, poorly tolerated by pregnant women, needs a previous patient preparation and presents issues with preanalytical stability and reproducibility (21,22). HbA1c, the most used measurement of chronic glycemia outside of pregnancy is easier to perform, does not need a previous patient preparation and is much less time consuming than the OGTT. However, the evaluation of HbA1c has its limitations such as: conditions that promote a reduction in the real value of HbA1c due to the reduction in the number of red blood cells, hemoglobin levels and hematocrit, conditions that increase the real value of HbA1c (21) and the period of pregnancy in which it is performed, being lower in the first trimester and around 0.5% lower at the 14^th^ week (22).

However it is important to emphasize that the accuracy of HbA1c as a screening tool in pregnancy has been studied in recent decades and the results are inconsistent (22–29). Thus, it would be necessary to establish reference values according to different ethnic populations before recommending the universal use of HbA1c for the screening of GDM (28). Khalafallah and cols. (21) aiming to compare HbA1c levels with glucose values on the 75g OGTT for the screening and diagnosis of GDM, found a HbA1c cut-off point of 5.4% associated with GDM, with a negative predictive value of 91.0% and a specificity of 95.0%. Similar results were also obtained when a cut-off point of HbA1c > 5.1% was used, with a sensitivity of 55% and a specificity of 80.0%, which was also the cut-off point we found (21). In our study, the cut- off point of HbA1c > 5.1% had higher sensitivity (70.9%) and lower specificity (71.6%) than this study.

Studies with small number of patients revealed an increase in inflammatory markers such as TNF-alpha (30) and CRP (31) in women with GDM which was not found in our study, maybe because the prevalence of obesity was small among those patients. In a study conducted in Austria, evaluating women with and without GDM an increase in CRP levels in pregnant women with GDM was found only at 37^th^-38^th^ weeks gestation but not at 24^th^-28^th^ weeks (32), the time frame we conducted our study. Cytokine levels fluctuate during the gestational trimesters, being the first and third trimesters characterized by a pro-inflammatory state and the second trimester by an anti-inflammatory state.

There was a correlation between TNF-alpha values, maternal BMI and CRP levels with placental weight in our study. This correlation, although weak (rho < 0.30), can point to a relationship between BMI with TNF-alpha and CRP that can generate damage to the placenta due to their inflammatory actions independent of glycemic levels. Retnakaran and cols. (31) performed a study with 180 healthy pregnant women who underwent an OGTT at the end of the 2^nd^ and beginning of the 3^rd^ trimester and found higher CRP levels in patients with normal OGTT and overweight and a correlation between CRP and prepregnancy BMI.

In our study, placental weight was an independent predictor for the presence of any of the following APOs (LGA, macrosomia, preterm birth and need for neonatal ICU admission) with a cut-off point of ≥ 610g. Studies performed with pregnant women having GDM indicate that they could have greater placental weight in relation to pregnant women having GDM (33,34), which we did not find in our study. In a study performed in Tanzania (35), in Sub-Saharan Africa, with 6,579 pregnant women, younger and with lower BMI than those patients enrolled in our study, low placental weight was associated with an increased risk of APOs. Possibly, a poorer nutritional status and the presence of infectious diseases in that population could have contributed for these results. Disruptions in placental growth can have long-term consequences on perinatal and childhood health and have been associated with adverse obstetric outcomes (intrauterine growth restriction and preeclampsia, maternal disease), perinatal mortality and morbidity, as well as impairment in childhood growth and development (10,35). A Romanian study (33), with pregnant women between 24-28 weeks of gestation showed that the placentas of six out of 13 GDM patients presented micro and macroscopic alterations. Macroscopically, the most frequent pathological changes found were larger placental size, volume and weight. It was also observed that, in the total sample, the presence of preterm birth was related to higher levels of fasting glycemia and CRP. The Camden Study conducted in USA, including 520 pregnant women with normal OGTT, showed that during pregnancy, higher levels of CRP were related to APOs, such as prematurity, only in lean women (BMI < 25) (36).

Some limitations of this study must be addressed such as the small number of patients and also the pregestational weight that was self-reported by the patients, which could generate some “bias”. We also correlated the presence of one or more APOs among those that were evaluated, with placental weight; therefore, we do not know which outcome had the greatest statistical strength this correlation.

In conclusion, in our study, some predictors of GDM are modifiable such as high BMIs and HbA1c levels. HbA1c values ≥ 5.1% at 24-28 weeks gestation were found to be associated with GDM, but we cannot hypothesize that this test could be used as a tool for GDM screening or diagnosis, because in our study, the sensitivity and specificity were low. A placental weight ≥ 610 grams was found to be associated with APOs, and consequently its weight should be monitored through ultrasound evaluation during the whole pregnancy as part of prenatal care routine to stratify risks for APOs. Further prospective studies with larger number of participants are necessary to confirm if this placental cutoff weight is associated to APOs for all pregnant women or just for those with GDM.
